# Editorial: Embedding current trends and innovative pedagogies in education in oral health: advancing educational practices and research

**DOI:** 10.3389/froh.2025.1569865

**Published:** 2025-02-19

**Authors:** Melanie Nasseripour, Angelo Leone, Ana Angelova Volponi, Bo Danielsen, Jonathan San Diego, Ana Estela Haddad

**Affiliations:** ^1^ Centre for Dental Education, Faculty of Dentistry, Oral and Cranio-Facial Sciences, King's College London, London, United Kingdom; ^2^ School of Medicine and Surgery, University of Palermo, Palermo, Italy; ^3^ Centre for Craniofacial and Regenerative Biology, Faculty of Dentistry, Oral & Craniofacial Sciences, King’s College London, London, United Kingdom; ^4^ School of Oral Health Care, University of Copenhagen, Copenhagen, Denmark; ^5^ Universidade de São Paulo, Sao Paulo, Brazil

**Keywords:** education, innovation, oral health, pedagogy, technology

**Editorial on the Research Topic**
Embedding current trends and innovative pedagogies in education in oral health: advancing educational practices and research

## Introduction

In the ever-evolving field of healthcare education, staying ahead of emerging trends and embracing innovative teaching methods is crucial for preparing the next generation of oral health professionals. As dentistry and oral health advance with technological breakthroughs and shifting patient needs, educators must continually refine their teaching strategies. This special issue explores these evolving educational approaches, showcasing their impact on both classroom instruction and oral health research.

## Current trends in oral health education

Digital technology is transforming education across many fields, and oral health education is no exception. Innovations like telehealth, artificial intelligence (AI), and simulation-based learning are changing how students acquire knowledge and develop clinical skills.

Furthermore, there is a increasing emphasis on interdisciplinary collaboration and community-based learning. These approaches prepare students for the complexities of real-world healthcare by fostering teamwork with professionals from other fields and engagement with diverse communities.

By extending learning beyond traditional clinical settings, they gain a comprehensive perspective on patient care.

## Innovative pedagogies in focus

Alongside technological advances, educators are also exploring new teaching methods that promote deeper understanding and critical thinking. One such approach, competency-based education (CBE), is gaining popularity in oral health programs. Unlike traditional models that emphasize time spent in the classroom, CBE focuses on mastering specific skills, ensuring that students are fully prepared to deliver high-quality care upon graduation.

Another promising strategy is the flipped classroom model, where students review course materials outside of class and use class time to practice skills and engage in more interactive learning activities. This approach creates a more collaborative environment and allows students to take a more active role in their learning.

## The role of research in advancing education

Oral health education continues to evolve, and research is crucial in guiding these changes. Evaluating and refining teaching methods through education research ensures that innovations are effective in improving student learning and clinical outcomes. This special issue invites contributions that examine the impact of these new approaches on student success, their relevance to real-world practice, and the experiences of both educators and learners.

Integrating research into the curriculum is equally essential. Engaging in research helps students develop critical thinking skills and learn to apply evidence-based solutions to problems in oral health. By doing so, they not only enhance their educational experience but also contribute to the broader body of knowledge in the field immediately and throughout of their lifetime.

## Special issue focus

The special issue highlights the following key points:

There is a critical need for e-learning platforms to address not only the academic requirements of students but also their psychological well-being (Zahid and Agou).
The virtual learning cannot fully replace the benefits of in-person instruction. Simulations and virtual patients can be used to establish clinical learning outcomes prior to the expected application of chairside learning (Meng).
Blended learning, which combines in-person and online learning offers students greater flexibility in accessing and engaging with learning materials, allowing them to learn at their own pace. There is a need to develop a more engaging and interactive online learning environment that rationally blends both online and face-to-face instructions, incorporating elements like group discussions, peer interactions, and virtual patient cases can help capture the collaborative nature of traditional in-person learning (Nasseripour et al.).
Both students and educators require support in developing essential, transferrable skills, including time management, proficiency in using AI tools for education, and general computing skills among many other skills. Strengthening these skills will enhance their ability to adapt to evolving learning and teaching methods (Byrne and Glasser).

For educators, digital and pedagogical literacy, particularly the ability to embed technology into teaching practice, remains essential. Even with a return to on-campus learning, maintaining a balance of synchronous, asynchronous, online, and in-person instruction offers valuable benefits. Developing these skills ensures educators can effectively navigate and enhance this blended approach to teaching.

Curriculum and pedagogy must continuously evolve to adapt and keep pace with advancements in:

• Knowledge
• Technology
• Understanding of how students learn, and the evolving role of oral health graduates
• The diverse needs of the communities they serve
• Interprofessional collaborative practice

Innovative teaching methods not only enhance student learning but also foster teamwork among students and educators, improve feedback quality and mechanisms, reduce costs and promote culture of continuous learning. Additionally, they can help alleviate the stress that oral health students face as they prepare to enter the job market, a significant concern for many (Babadi et al., Miao et al.).

Dialogue with students and staff to explain the reasoning behind any new pedagogy, new technology adoption is essential to the success of such endeavours (Hasan and Jones).

When innovations are introduced, a review of previous approaches is required to determine what can be modified (reduced or removed), helping to maintain a manageable workload for both students and educators. Thoughtful planning of this transition by phasing in new approaches and phasing out the outdated ones, is crucial to sustaining goodwill in adopting innovation.

For innovations to be successful, educational institutions must be willing to rethink teaching and assessment approaches, effectively manage the innovative changes, and provide the necessary physical and technological resources (Liu et al.).

Time or lack thereof is repeatedly reported as a barrier to implementing innovative pedagogies. Institutions must value the time it takes to develop and implement innovative approaches to oral health education (Nasseripour et al.).

## Conclusion and next steps

As oral health education continues to evolve, integrating emerging trends and innovative teaching methods in the curricula is essential. The contributions in this special issue offer valuable insights for educators, researchers, and practitioners who are working to transform educational practices. By fostering a collaborative environment that prioritizes both learning and research, we can ensure that future oral health professionals are well-equipped to meet the needs of an ever-changing healthcare landscape.

We encourage readers to explore the articles in this issue, reflect on how they might apply these ideas in their own teaching, and consider how collaboration across disciplines can further enhance the learning experience. Together, we can shape the future of oral health education and prepare the next generation of practitioners for success.

